# BiologicalNetworks - tools enabling the integration of multi-scale data for the host-pathogen studies

**DOI:** 10.1186/1752-0509-5-7

**Published:** 2011-01-14

**Authors:** Sergey Kozhenkov, Mayya Sedova, Yulia Dubinina, Amarnath Gupta, Animesh Ray, Julia Ponomarenko, Michael Baitaluk

**Affiliations:** 1 San Diego Supercomputer Center, University of California San Diego, 9500 Gilman Drive, La Jolla, CA, 92093, USA; 2 Skaggs School of Pharmacy and Pharmaceutical Sciences, University of California San Diego, 9500 Gilman Drive, La Jolla, CA, 92093, USA; 3 Keck Graduate Institute, 535 Watson Drive, Claremont, CA, 91711, USA

## Abstract

**Background:**

Understanding of immune response mechanisms of pathogen-infected host requires multi-scale analysis of genome-wide data. Data integration methods have proved useful to the study of biological processes in model organisms, but their systematic application to the study of host immune system response to a pathogen and human disease is still in the initial stage.

**Results:**

To study host-pathogen interaction on the systems biology level, an extension to the previously described BiologicalNetworks system is proposed. The developed methods and data integration and querying tools allow simplifying and streamlining the process of integration of diverse experimental data types, including molecular interactions and phylogenetic classifications, genomic sequences and protein structure information, gene expression and virulence data for pathogen-related studies. The data can be integrated from the databases and user's files for both public and private use.

**Conclusions:**

The developed system can be used for the systems-level analysis of host-pathogen interactions, including host molecular pathways that are induced/repressed during the infections, co-expressed genes, and conserved transcription factor binding sites. Previously unknown to be associated with the influenza infection genes were identified and suggested for further investigation as potential drug targets. Developed methods and data are available through the Java application (from BiologicalNetworks program at http://www.biologicalnetworks.org) and web interface (at http://flu.sdsc.edu).

## Background

Public health initiatives increasingly recognize the importance of the cross-scale data integration, such as mounting a data-driven risk assessment of potential pandemic outbreak in specific geographical locations or discovering novel therapeutic approaches [1-6]. For example, to facilitate the study of the Influenza infection outbreaks [7,8], it is desirable to apply the systems biology approach that requires integration of heterogeneous data from various domains of knowledge: flight paths of migrating birds, animals and humans; virological aspects, such as the efficiency with which the virus can be transmitted from the infected subject; cellular phenomena, such as interaction of viral proteins with surface receptors in the inner and outer respiratory tracts of hosts; phylogenetic properties of viral strains and viral proteins; structural properties of proteins; and molecular interactions of host and virus proteins to each other and small molecules [9-11]. Thus, there is a need in the integration system able to integrate heterogeneous biological and clinical data and enable cross-domain and cross-scale analyses of those data.

Experimental data on host-pathogen interaction are distributed throughout many heterogeneous data sources. Among the integration systems enabling studying host-pathogen interactions at multi-level scale are PHI-base [12], PHIDIAS [13], PIG [14], IVDB (Influenza Virus Database) [15], and the NCBI Influenza Virus Database [16]. In these resources, data sources are integrated mostly through URL links. Despite the active research in the field, most of the published data concerning host-pathogen interactions [17-28] are not available for the study in the concert with other data: they can be accessed only as supplemental tables to the papers and at best visualized using the network visualization and navigation tools, such as Cytoscape [29], GenMAPP [30], GeneSpring (Agilent). These solutions, however, do not allow integration of orthogonal types of data, such as 3D protein structures or sequences of gene regulatory regions, for example. They also do not allow phylogenetic, orthologous or phylogeographic analysis that is necessary, considering the fact that the detail experimental analysis of host-pathogen interactions for each of the existing, emerging and reemerging pathogens is not feasible.

At the same time, existing link-based integration systems, such as Entrez [31], Ensembl [32], or BioMart [33], provide limited capabilities for analysis of host-pathogen interactions and pathways specifically. While most heterogeneous data integration systems, or warehouses, are either domain-specific--for example, STRING [34], GeneCards [35], or PharmGKB [36] deal with genomic data exclusively--or do not allow sequence search and annotation, for example, ONDEX [37], BIOZON [38], or BNDB [39].

In this paper, the approach at cross-scale data integration to study host-pathogen interactions is proposed and demonstrated on a study of the *Influenza *infection. The proposed system is an extension of the previously developed BiologicalNetworks [40,41] and IntegromeDB [42]. It represents a general-purpose graph warehouse with its own data definition and query language, augmented with data types for biological entities. Developed methods and implemented solutions for the integration, search, visualization and analysis of host-pathogen interaction data are available through the BiologicalNetworks application http://www.biologicalnetworks.org and web interface http://flu.sdsc.edu; Demo page: http://flu.sdsc.edu/examples.jsp.

## Methods

### System

The architecture of the system, data integration and mapping procedures, database schema, ontology model and data query engine are described in detail elsewhere [42]. Therefore, only brief description is provided here. Data integration and mapping to the internal database is fully automated and based on Semantic Web technologies and Web Ontology Language (OWL) http://www.w3.org/TR/owl-ref. The IntegromeDB [42] internal database schema is RDF-compatible (Resource Description Framework; http://www.w3.org/RDF/); that is, it stores biological data in an RDF-compatible format, the standard format of the Semantic Web [43]. The database architecture and database schema are provided at http://www.BiologicalNetworks.net/Database/tut0.php. The ontology is available as an OWL file at http://flu.sdsc.edu/bionetsonto.jsp.

### Data

The full list of integrated databases and statistics are provided at http://www.biologicalnetworks.net/Database/tut5.php[42]. To enable research on host-pathogen interactions, in addition to previous integrated data on genome and protein sequences, gene expression and regulation data, protein-protein and protein-DNA interactions [42], the following data were integrated (Table 1):

**Table 1:** Integrated data for the Host-Pathogen interaction studies

	Host	Pathogen	Host-Pathogen
**Genome strains**	-	22949	11843 (H)

**Genome Sequences (complete genomes)**	354	3994	-

**Protein Sequences**	99117	58218	-

**cDNA library Sequences (+conditions, tissue sources)**	79431	47175	-

**Gene expression**	12983	-	29

**3D structures**	135819	380	-

**Protein- protein interactions/Reactions/Relations**	> 200 000 000 in more than 1000 organisms	650	250

**Phylogeny**	9 phylogenetic tree libraries from PhyloFacts database [44]		

**Virulence**	Statistics on confirmed Human cases of Influenza reported to **WHO**		

**Epidemiology**	Infection occurrence, pathogen culture sites, dates, migration data vectors, movement data of infected individuals for 113 countries, 1605 locations		

• P*hylogenetic trees *that connect host and pathogen proteins/genes with orthologs/homologs in model organisms (with their molecular sequence, structure, expression and interaction data). These data were obtained from PhyloFacts database [44].

• *Literature*-curated information on physiological effects of pathogen infection in experimental systems (including cell cultures and *in vivo *models)

• V*irulence *data about mortality/morbidity information related to isolate and incidence, isolated organism's sequence data (from WHO statistics)

• *Epidemiological *data on infection occurrence, pathogen culture sites and dates, migration data of vectors, past movement data of infected individuals, etc. (from NCBI Influenza Virus Resource).

Additional experimental data sets on host-pathogen interactions integrated into our system include human interactomes used by Influenza virus, HIV, HCV, dengue virus and West Nile virus (WNV) (based on the results reported in [17,22-28]).

### Data access

**Figure 1:**
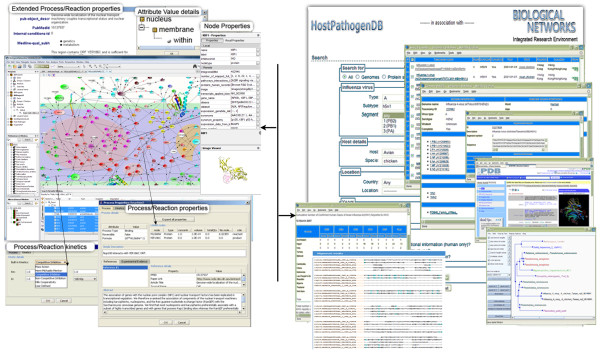
**BiologicalNetworks and web interfaces for host-pathogen interaction studies**.

The web page http://flu.sdsc.edu (Figure 1) provides genomes/pathogenic strains searches by keywords and genomic and protein sequences, statistics on integrated data by category and data source, information relating to retrieved properties by data sources for each gene/protein that can be accessed from the query result page, and data inconsistencies in public data. The web site was designed primarily for the purpose of giving the user an opportunity to quickly search for phylogenetic relations among sequence strains and perform at integrated data rather than to provide complex data analysis capabilities, which are implemented in the BiologicalNetworks application, which can be downloaded at http://www.BiologicalNetworks.org (Figure 1).

To integrate the user's data into the system, the web page http://flu.sdsc.edu/integration.jsp (see Section 'Integration of user's data' below) is provided. The data will become public, but unless curated by the data administrators, will remain "tagged" as 'uncurated' under the contributor's name. The user's data integration procedure consists of 3 simple steps: 1) User registration, 2) Data Mapping and 3) Data integration (see Section 'Integration of user's data' below). To be integrated, the data needs to be in the table format.

## Internal Data Model and Data Structures

**Figure 2:**
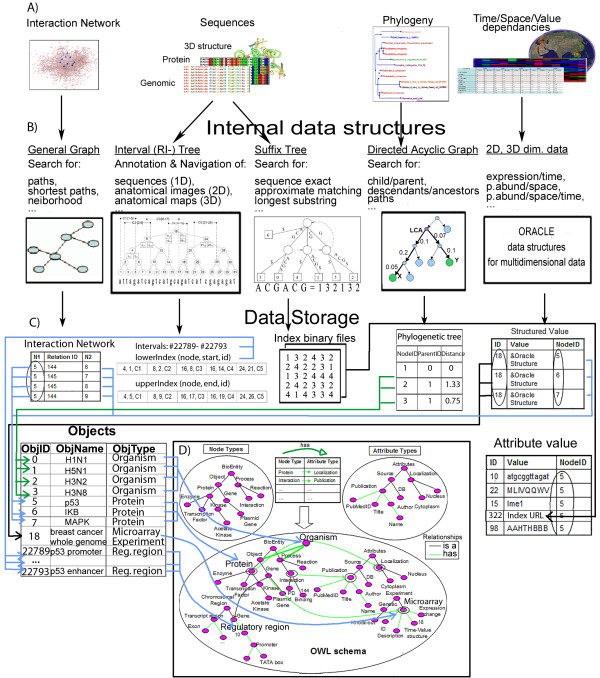
**Integration of diverse genomic and meta-genomic data in BiologicalNetworks for the host-pathogen interaction studies**. **(A) **Four main data types (*i.e*. Graphs, Sequences, Trees and 'Histograms') representing diverse range of biological data are integrated in the BiologicalNetworks. **(B) **Internal data representation of main four data-types. Interaction/Relation networks are stored as general graphs, Genomic/Protein/3D-structure sequences- as interval (RI-) trees and Suffix trees, Ontologies/Phylogenies as DAGs (Directed Acyclic Graph), Histograms as Oracle structures for multidimensional data. **(C) **Data storage schema, internal data tables and binary files for storing and integrating diverse data types. **(D) **BiologicalNetworks Ontology reflects the current knowledge of the domain, taking information from many ontologies provided by OBO consortium at http://www.bioontology.org.

The internal schema of the BiologicalNetworks database is shown in Figure 2. Four orthogonal types of biological data--graphs, sequences, histograms, and tree structures--are integrated to enable multi-scale data analysis for the host-pathogen studies. In the process of integration, all external data types (Figure 2A) are transformed into graph and tree data structures (Figure 2B): one-dimensional sequence data (*e.g*. sequences) into interval trees; two- and three-dimensional data (*e.g*. images) into R-trees. To keep the number of the index structures small, a single interval tree is created per chromosome instead of per annotated DNA sequence regions, and the images of the same resolution are referenced with respect to the same coordinate system and placed in a single R-tree. The examples of operations on RI-trees that are used in BiologicalNetworks for *Navigation *and *Annotation *of multiply overlapping gene regulatory regions, protein binding regions, disease and geographical maps are provided in the Additional File 1, Section S1.1.

Nodes of the interval and R-trees (Figure 2C) are connected to ontology nodes (Figure 2D) through the internal Objects and Attribute values tables that are in turn connected through BioNets Ontology http://flu.sdsc.edu/bionetsonto.jsp (Figure 2D). BioNets ontology consists of three parts: (i) the general-purpose Basic Ontology, which is the modification of BioPAX Level 2 ontology; (ii) manually (by the authors) mapped to the basic ontology 25 selected OBO ontologies (specified at http://flu.sdsc.edu/bionetsonto.jsp and provided in the Additional File 1, Figure S2); and (iii) 72 OBO ontologies that are mapped to each other and the selected 25 ontologies as provided by the OBO consortium. The basic ontology and mappings to the selected 25 ontologies are provided in the file basic.owl available at http://flu.sdsc.edu/bionetsonto.jsp under "MappinSuperClass" class and "sameAs" properties.

A new ontology can be introduced without modification of the BioNets ontology classes and through 'ontology mapping' [43]. For example, for a SequenceOntology that maps a class *Gene *'SO:12345', a new class 'mappingSO:012345' will be generated using 'same_as' relation. More detailed information on BioNets ontology is provided in the Additional File 1, Section S1.2.

Versatile suffix tree structures (Figure 2B) are used to solve a variety of sequence-based problems, such as exact and approximate matching, database querying, and finding the longest common substrings [45]. A variety of efficient in-memory suffix tree construction algorithms have been proposed [45-50], that are scalable with extremely large (for example, the human and mouse genomes are approximately 3 Gbp and 2.5 Gbp long, respectively) sequences. External biological sequences are transformed to internal suffix tree structures, using TRELLIS algorithm [51]; sequence search operations such as exact sequence search, best match, and longest substring are allowed. Suffix tree representation of genomic/protein sequences is stored as indexed binary files and is mapped to the database sequence objects as property values (Figure 2B and 2C (right)).

## Data Querying

As different categories of data are added to the system, it becomes critical to have an augmented (internal) query language that provides constructs (operators and functions) to search, manipulate and query the data. The developed for this purpose the *BioNetQL *query language is used behind the user interface in the BiologicalNetwork application. It can be also used by the users accessing the database directly through the API; for example, our database is extensively used through the BioNetQL API in the CAMERA metagenomic project [52]. The syntax of BioNetQL and its distinction from SQL and SPARQL are considered in the Additional File 1, Section S1.3.

To enable systems-level study of host-pathogen interaction, in addition to the query capabilities described previously [42], a number of new methods were implemented that now allow answering the specific questions concerning host-pathogen interactions. For example, the following questions: (1) What is the evolutionary distance between two specified genome sequences? (2) Which genome sequences are within the specified evolutionary distance from a given genome? (3) What is the probability of a given protein/gene sequence to be virulent? and (4) What is the probability of a protein of a given 3D structure to be virulent? Evolutionary distance was chosen to rationalize the information integration scheme of our database, because virus properties, such as virulence, infectivity, host-specificity, geographic locations, morbidity in an epidemic, or host-specific reactions are related by evolutionary lineages [53].

**Figure 3:**
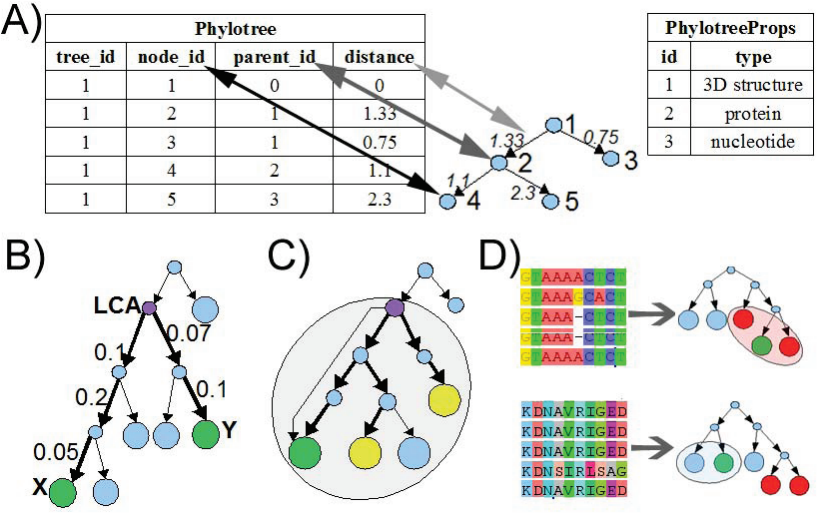
**The rationale of phylogenetic querying in BiologicalNetworks**. **(A) **Phylotree database table. **(B) **Calculation of the evolutionary distance between two strains *X *and *Y*. **(C) **Find genome strains with the range of evolutionary distance. **(D) **Estimation of the probability for node *Y *to be virulent by multiple sequence analysis of it's neighbors.

To address the first question, the database can be searched for evolutionary distances between two specified genome sequences of different species/strains/isolates, executing the following three queries (Figure 3A, B): (1) reconstruct the PhyloTree containing both sequences; (2) find the least common ancestor (LCA) for two sequences; and (3) find the sum of distances from each of the two sequences to the LCA.

To find the answer to the second question--Which genome sequences are within the evolutionary distance L_max _from a given genome X?--the following queries need to be executed (Figure 3C): (1) reconstruct the phylogenetic tree containing X; (2) find the ancestor A of the node X that is no farther than L_max _from X; and (3) in the ancestor A rooted sub-tree, find the nodes at the distance L_max _from X.

To predict the virulence of a species/strain/isolate by its gene/protein sequence, the published method for virulence evaluation of low and high pathogenic avian influenza LPAI and HPAI [53] was implemented in the system. Using this method, it was shown that HPAI strains in addition to phylogenetic grouping exhibit grouping by geographical region [53]. The following sequence of queries allows applying the method (Figure 3D): (1) recalculate/modify the phylogenetic tree based on multiple sequence alignment; (2) find the nodes nearest to a given node X; and (3) calculate the probability for the node X to be virulent if its neighbors are known to be virulent or not virulent.

Similarly, the probability of a protein of a given 3D structure to be virulent can be estimated, using the phylogenetic tree built based on 3D structure multiple sequence alignment (Figure 3D).

To enable the aforementioned queries, a number of bioinformatics methods were implemented in BiologicalNetworks both in house and by the others to: reconstruct the phylogenetic tree, recalculate/modify the phylogenetic tree, for multiple sequence alignment [54,55].

Also, for identifying phylogenetically conserved transcription factor binding sites in the gene regulatory regions, the method [56] was applied to the promoter sequences ( the region from -500 to +500 bp relative to the transcription start site) of all integrated genes, using known binding sites that have been integrated in the IntegromeDB [42]. Identified gene pairs and their conserved binding sites were integrated into the system. In addition to the queries by keywords, this data can be queried by sequences (the examples are given in the Application section). The implemented in BiologicalNetworks approach to the sequence querying is discussed in detail elsewhere [42].

## Results and Discussion

**Figure 4:**
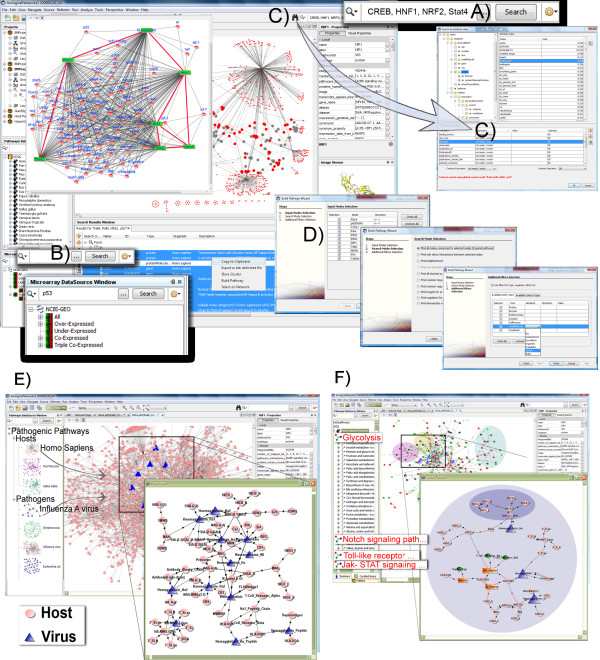
**BiologicalNetworks data querying interface for host-pathogen interaction studies**. **(A) **Keyword and multi-word search. **(B) **Specialized search. **(C) **Comprehensive search by attributes. **(D) **Build Pathway Wizard. **(E) **Composite network of molecular pathways active in Influenza A-infected dendritic tissue implicated by genomic expression data. **(F) **Extracted graph of flu virus antagonist effects on Glycolysis metabolism in dendritic cells.

The severity of flu pandemic outbreaks, including the recent one of the swine-origin H1N1 influenza virus, and widespread resistance to the existing antiviral drugs demand for new therapeutics targeting host factors. Identification of host genes involved in the virus-host interactions is the first step towards developing such therapeutics [17]. In this work, we attempted to identify these genes, analyzing the broad spectrum of publicly available data on influenza viruses and infections, using the BiologicalNetworks application (Figure 4A-D).

To find the potential therapeutic targets in the host, we first identified the genes that were differentially expressed in mouse and human in response to the influenza infection and interacted with the virus and to each other. Then, using the constructed interaction network, we studied the proteins that directly interacted with the virus and co-expressed genes. Sub-networks that were induced/repressed at different stages of the influenza infection were also analyzed. In the result of analysis of co-expressed genes and transcription factor binding sites in their promoters, 118 genes were identified as potential candidates for further investigation; after filtering the genes that are known to be associated with influenza infections, 7 genes were obtained.

Using the influenza viruses as an example, in the following four sections we demonstrate and discuss different types of host-pathogen interactions that are available in BiologicalNetworks. Since at the moment of the analysis, the data were available for only two influenza proteins, neuraminidase and hemagglutinin, the provided below analysis was narrowed down to these two viral proteins. All discussed queries are provided in detail in the Additional file 1, Section S1.4.

The last two sections show additional capabilities of the system, phylogeographical analysis and integration of the user's data, following by the comparison with other systems designed for studying host-pathogen interactions.

### Building the influenza virus-host interaction network

To construct the interaction network for further study, BiologicalNetworks was first queried for the host proteins interacting with the virus and localized in nucleus or cytoplasm (Query 1, Additional File 1, Section S1.4). This query returns the set of pair-wise interactions in the form of a graph. Then, BiologicalNetworks was searched for the genes that were differentially expressed and related to the influenza infection in the microarray experiments (Query 2 Additional File 1, Section S1.4). Since different experiments have different number of time points and conditions, data from every experiment were normalized (for details see Additional File 1, Section S1.4).

Finally, for the discovered genes, we extracted all known interactions among host proteins. Thus, among 3,972 differentially expressed genes in mouse, ~12,000 interactions were found for the proteins implicated in the influenza expression data set. After narrowing the search to the curated interactions from HPRD and BIND databases, we obtained the resulting network consisting of 4,592 interactions among 1,950 molecules (Figure 4E). Due to limited data available in human, the network for human was much smaller and contained only 413 influenza-human and human-human interactions. Now, the constructed networks can be examined in detail.

### Analyzing sub-networks

In this section, we demonstrate the analysis of sub-networks and individual interactions in the interaction network.

First, we were interested in identifying sub-networks that were significantly induced or repressed relative to randomly selected sub-network [57]. Six such sub-networks were found; they overlapped and each consisted of about a hundred interactions. The pathways found in these sub-networks were consistent with a large-scale response of complex molecular pathways to the viral infection. Thus, genes involved in the interferon-response pathway were induced, owing to the immune response to viral infection. Nearly all genes involved in the Jak-STAT interferon-response signaling pathway and apoptosis-related genes were activated; whereas the genes involved in the growth factors (IGF and connected pathways), cell-cycle and translation-related pathways (CDKN and connected pathways) were repressed.

Second, six sub-networks were combined into a single fully-connected network (Figure 4F); that is, all interactions that did not belong any of the sub-network were excluded. In this network, we looked for the sub-networks that were significantly perturbed at early, middle or late stages of the influenza infection. As it was expected, "early" sub-networks contained pathways of the general immune response, whereas "middle" and "late" sub-networks - pathways specific for the infection. The genes involved in the pathways significant for early, middle and late stages are shown in Figure 4F and colored according to the stage. Further, we looked at the GeneOntology terms for the genes in the pathways that were perturbed at early, middle or late stages of the influenza infection. The following biological processes from GeneOntology were identified: immune response (*p-value <*1.0e-6), proteolysis and peptidolysis (*p-value < 1.0e-5*), lipid transport (*p-value < 0.001*), and complement activation (*p-value < 0.01*).

### Studying individual genes

To study individual genes, we used the constructed in the previous section network of significantly induced/repressed genes in the influenza infection (Figure 4F). For example, one can search for the genes that directly interact with the viral proteins and are known to be up- or down-regulated in human or mice (Query 3 in Additional File 1, Section S1.4). Thus, among the down-regulated genes were the genes of the immune response (Toll-like receptors TLR1/TLR2 and interleukin), interferon-regulated genes (interferon-induced protein with tetratricopeptide repeats 2 (IFIT2) and vipirin), and the other genes involved in defense, inflammatory response and intracellular signaling pathways, including chemokine, apoptosis, MAPK, Notch, Jak-STAT, T-cell receptor, complement and coagulation cascades--pathways and genes are known to participate in the viral response and recruited by the virus for the entry [17,58,59].

Further, we decided to focus on co-expressed genes. Such an analysis can give us host genes that can be potential targets for anti-viral drugs. We selected from the mouse network 45 co-expressed genes that were also differentially expressed in response to the influenza infection (Query 4 Additional File 1, Section S1.4). Since our database contains information on transcription factor binding sites, both experimental, predicted and conserved, we used that information to extend the network beyond the reported interactions. In the promoters of 45 co-expressed genes, we searched for binding sites that were conserved in the three species, *Homo sapiens*, *Mus musculus *and *Rattus norvegicus*. Such sites were found in 7 genes: SFRS11, SFRS1, FMNl2, LEPROT, NICN1, FHOD2, f3-contactin; if one is interested, the binding sites can be searched by sequence (Query 6 in Additional File 1, Section S1.4). Identified conserved binding sites were regulated by 73 transcription factors, including CREB, HNF1, NRF2, FOXP3, and factors from Pax, Gata and Stat families.

### Potential drug targets

Which of the identified 7 co-expressed genes with phylogenetically conserved binding sites and their 73 potential transcription factors were not previously reported as associated with the influenza infection? The search (Query 7 in Additional File 1, Section S1.4) gave us 7 such genes/proteins: NFE2L2 (NRF2), FOXI1, SMAD6, HOXA3, SFRS11, GRAP, and AMPD1.

Based on available information, 5 genes, SMAD6, GRAP, HOXA3, NFE2L2, and AMPD1, might be suggested to be further investigated as drug targets in the influenza infection. Thus, SMAD6 is known to be involved in immune response (GO:0006955), signal transduction and transcriptional modulation of multiple signaling pathways, including BMP (GO:0030509) and TGF-beta receptor-signaling (GO:0030512). GRAP is involved in activation of the T-cell antigen receptor (TCR) signal transduction pathway [60], Ras protein signal transduction (GO:0007265) and cell-cell signaling (GO:0007267). HOXA3 transcription factor may be required for the induction of pathogen-response genes in humans as it was shown that *S. aureus *infection induced a number of HOX genes that modulated the NF-κB -dependent transcription and exerted this function redundantly [61].

AMPD, or AMP deaminase, is an enzyme that converts adenosine monophosphate (AMP) to inosine monophosphate (IMP), freeing an ammonia molecule in the process. Deficiency of this enzyme is a common cause of myopathy and rhabdomyolysis (the rapid breakdown of skeletal muscle). The influenza infections are known to be a cause of rhabdomyolysis, including seasonal [62] and recent H1N1 [63].

NFE2L2 gene codes the transcription factor NRF2 that is a known master regulator of the antioxidant response [64]. By inducing genes involved in combating oxidative stress that results in inflammation, neurological diseases, and renal disease, NRF2 protects body from a variety of oxidative stress-related complications. NRF2 activators have been studied as cancer [65] and diabetes drugs [66]. Our research shows that NRF2 might be considered a therapeutic candidate in the influenza infection as well.

### Adding phylogeography

**Figure 5:**
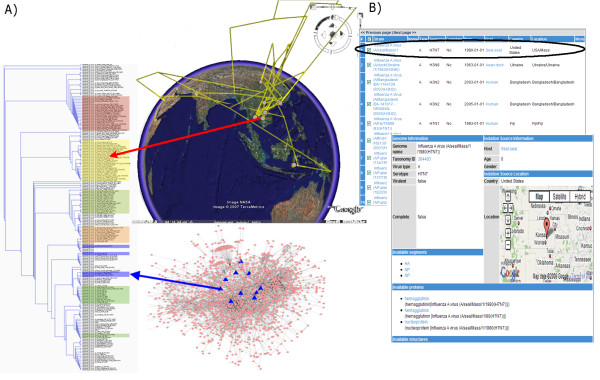
**Visualization of phylogeographic data in Influenza virus study project**. **(A) **Influenza virus clades are labeled by corresponding geographical region and visualized phylogenetically on **GoogleEarth**. Every spot on the globe corresponds to a particular strain of Influenza outbreak. Each leaf of the phylogenetic tree represents an influenza strain, and is located in the place where corresponding virus genome was found. This representation allows easily identify approximate location of the root node. The host-virus interaction network corresponds to one specific virus study. **(B) **Example of the genome detail page, with map showing location where a particular virus strain was isolated.

BiologicalNetwork allows phylogeographical analysis of a pathogen(s) strains/isolates. In particular, host-pathogen interaction networks for two or more pathogen genomes can be compared, using both phylogenetic and geographical data (Figure 5). Phylogeography seeks to explain the molecular evolution, genealogy and migration of species [54]. The strains in question are more likely to have similar pathogen-host interactions (and virulence properties) if they originated from the same geographical location and have relatively small phylogenetic distances [53,67]. Using phylogeographical approach, the closest phylogenetic neighbor for a new sequenced pathogen strain can be found, and its probability to be virulent can be estimated. The phylogeographical methods implemented in BiologicalNetworks are described in the section Methods and Demonstration page at http://flu.sdsc.edu/examples.jsp. Together with capability to integrate new phylogeographic data, these methods make BiologicalNetworks a unique among other integration systems. BiologicalNetworks allows visualizing and comparing host-pathogen networks in respect to the pathogens phylogenetic distance and geographic origin (Figure 5).

### Integration of user's data

**Figure 6:**
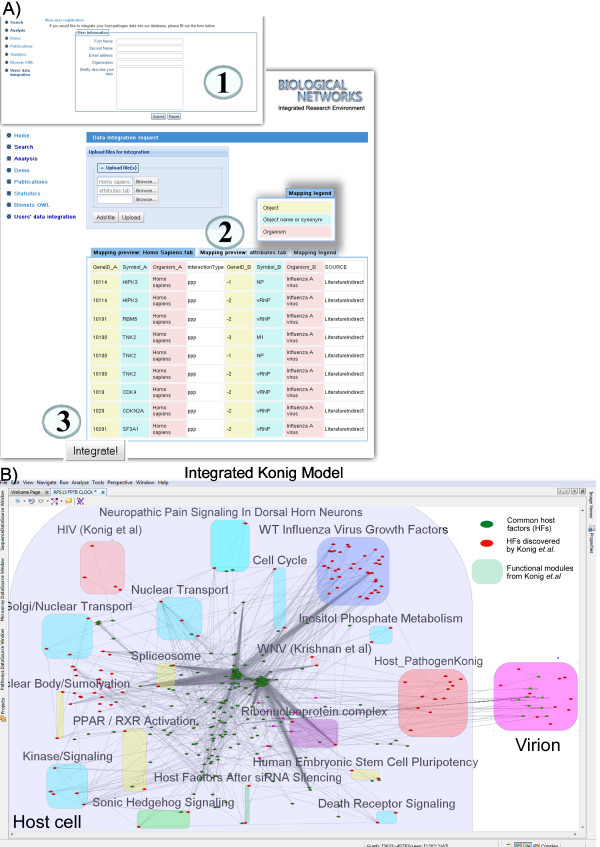
**User's Data integration**. **(A) **User's Data Integration page allows integrate host-pathogen data which is interesting for particular user, but we didn't integrated. The integration procedure consists of three easy steps: 1) User registration, 2) Mapping of the data and 3) Integration of the data. We have integrated supplementary data provided in the studies of Konig *et. al. *[17] and others [21-28]. **(B) **Meta-network model of the Konig *et. al*. data integrated (in **A)**) into the IntegromeDB database and available to be analyzed in concert with wealth of other host-pathogen data available in BiologicalNetworks [40-42]. Host factors that were already present in our database and known to be related to viral response are colored green, whereas host factors newly discovered by Konig *et. al*. as being related to viral infection are colored red. Supplementary data from Konig *et. al*. integrated into our system are represented as colored meta-nodes (boxes): cyan- functional groups, yellow- molecular complexes, green - factors over-expressed after siRNA silencing, purple- biochemical complexes that are required by different RNA viruses, blue- host proteins confirmed to be required for wild-type influenza virus growth, pink -other virus experiments/publications (*e.g*. HIV, HCV, WNV, *etc*.) (see Supplementary data in Konig *et. al.*).

Any public or user's data in the table-format can be integrated into BiologicalNetworks automatically and studied together with other already integrated data. The user can do it at http://flu.sdsc.edu/integration.jsp (Figure 6A). We integrated host-pathogen interaction networks, pathways and all other data provided in the studies of Konig *et. al*. [17] and others [21-28]. Data from [17] are provided in 13 supplementary tables and contain: human cellular factors required for early-stage influenza virus replication, biochemical complexes that are required by different RNA viruses, host proteins confirmed to be required for wild-type influenza virus growth, groups of genes over-expressed after siRNA silencing, other virus experiments/publications (e.g. HIV, HCV, WNV, etc.), *etc*. After automatic mapping and integrating those tables, we were able to visualize them as an integrated meta-network model in BiologicalNetworks. Now this meta-network model can be studied in relation to the data it was generated from--for example, different modules described in different data tables in relation to each other (Figure 6B, colored boxes)--and in relation to the other integrated data. For example, asking the system on what is known about the data imported from [17] (BuildPathwayWizard function "Find Meta Models" with specified parameters can be used for that), genes/proteins in the network can be seen in connection to the papers they were co-cited (Figure 6B) and/or mentioned together in supplementary materials. Similarly, the nodes in the network can be analyzed for co-expression, expression in particular tissues/cell types, and other functional information, such as functional modules, protein complexes and canonical pathways. The model is available for exploration either from BiologicalNetworks application as Demo Project or from the web site http://www.biologicalnetworks.org.

The ability to integrate Supplementary data for host-pathogen studies and to represent it in the digital integrated form is an extremely important feature for reproducible and integrated research. Several journals including *Nature*, *PLoS*, *Cell *are working towards establishing the reproducible research standards for their publications. In addition to asking the authors to represent their data in standard formats (e.g. MIAME for microarray data, SBML/SIF for network data, *etc*.), Cell journal, for example, now asks the authors to accompany their publications with graphical abstracts. Presenting the data in the form of a digital integrated model (*e.g*. BiologicalNetworks project file that can be opened on any computer) instead of the graphical picture would be much more useful for researchers and our future work will be towards that direction.

### Comparison with other systems

We chose for comparison six resources: Cytoscape [29] and five resources developed specifically for studying host-pathogen interactions, PHI-base [12], PHIDIAS [13], PIG [14], IVDB (Influenza Virus Database) [15], and the NCBI Influenza Virus Database [16].

No two resources were similar by all 15 properties considered (Table 2). Most of the resources provide pathways and microarray data, however analysis and search of both types of data is provided only in Cytoscape and BiologicalNetworks. Phylogenetic analysis and sequence search are provided only in BiologicalNetworks and NCBI Influenza Virus Database. No resource except BiologicalNetworks is capable of analyzing regulatory regions, orthologous genes, 3D structural data, or dealing with phylogeographical data.

**Table 2:** Comparison of BiologicalNetworks/HostPathogen database with public host-pathogen interaction resources

	PHI-base	PHIDIAS	NCBI Influenza db	PIG	IVDB	Cytoscape	BiologicalNetworks/HostPathogenDB
Scalability*	no	no	no	no	no	no	yes

Data integration engine	no	no	no	no	no	no	yes

Interaction and Pathways data/analysis	yes	yes	no	yes	no	yes	yes

Chemicals/Drug Discovery	no	no	no	no	no	yes	yes

3D structure	no	no	no	no	no	no	yes

Sequence annotation/search	no/yes	yes/yes	yes/yes	no/yes	no/no	no/no	yes/yes

Phylogenetic analysis	no	no	yes	no	yes	no	yes

Regulatory regions analysis	no	no	no	no	no	no	yes

Orthology analysis	no	no	no	no	no	no	yes

Phylogeoraphy	no	no	no	no	no	no	yes

Microarray data/analysis	no/no	yes/no	yes/yes	yes/no	no/no	yes/yes	yes/yes

Web search/ Research environment	yes/no	yes/no	yes/no	yes/no	yes/no	no/yes	yes/yes

*Scalability to the number of integrated data sources.

Due to the graph-based data integration model and the Semantic Web technologies implemented in BiologicalNetworks [42], it is scalable in respect to the number of integrated resources and therefore allows integration of user's data--this is the absolute merit of the proposed system for studying host-pathogen interactions.

## Conclusion

BiologicalNetworks extensions for the host-pathogen studies enable diverse data in major human-disease systems to be subjected to efficient integrated analysis. The results show the utility of multi-scale data integration from large-scale human molecular-interaction, sequence and expression data to epidemiological and virulence data. The approach described allows information to be extracted that is not restricted to any one data type. Moreover, our analyses suggest how various host pathways act in response to viral infection, and serve as a large-scale window into the genomic response to Influenza and other respiratory infections. The pathways identified should provide insights into the mechanisms by which the host interacts with different pathogens, useful information about stage of disease, and selection of suitable targets for early diagnosis and treatments.

BiologicalNetworks has general purpose graph architecture and is data-type-neutral. Therefore, there is the prospect of further integration of data such as detailed clinical data that will enable clinical variables to be associated quantitatively with the activities of molecular pathways and processes. Capacity for integration gives our system a unique capability, the full potential of which will be realizable when a multitude of host-virus interaction data are available, so that similarities and differences between the interaction networks can be interrogated across the phylogenetic distance for more accurate prediction of the potential virulence of a newly isolated virus identified only by its sequence. We believe that the methods and tools we have implemented and described here will allow for the efficient dynamic integration and analysis of diverse data in other disease systems.

## Authors' contributions

SK, MS, AG and MB contributed to system concept. SK, MS, YD and MB implemented the system and performed major programming work. MB, JP and AnR contributed to data analysis. This work was coordinated by AG, AnR, JP and MB. MB and JP wrote the manuscript. All authors read and approved the final manuscript.

## Supplementary Material

Additional file 1 **Methods**. Detailed description of the methods and data types used in the BiologicalNetworks system for host-pathogen studies. Click here for file
