# Supplementary material for: BiologicalNetworks - tools enabling the integration of multi-scale data for the host-pathogen studies
Source: BMC Syst Biol. 2011 Jan 14;5:7. doi: 10.1186/1752-0509-5-7 (PMC3027118; doi:10.1186/1752-0509-5-7)
Supplement: Additional file 1 — Methods. Detailed description of the methods and data types used in the BiologicalNetworks system for host-pathogen studies. [file 1752-0509-5-7-S1.DOC]

Additional File 1


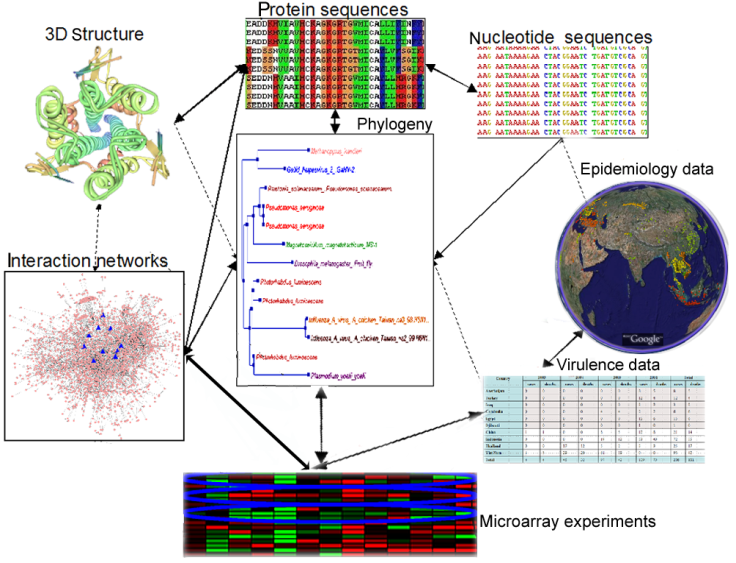


**S1. The rationale for the diverse data types integration in the Host-Pathogen interaction studies.**

**S1.1 Data structures used for the host-pathogen studies**

**RI-trees**

Before the data integration process external biological data types are transformed into various versatile graph and tree data structures (Fig. 2). Sequence data (genomic, protein, 3d structure (β-sheets, α-helixes, *etc.*)) together with the annotation data (binding sites, gene regulatory regions, *etc*) are stored in a collection of interval and suffix trees. For 1-dimentional data (e.g. sequences) interval trees are used and for 2D and 3D data (e.g., anatomical image regions, geographical maps) a collection of R-trees is used. Simple techniques are used to keep the number of the index structures small: a single interval tree is created per chromosome instead of per annotated DNA sequence regions; all anatomical (brain, heart, *etc.*) and geographical images of the same resolution are referenced with respect to the same coordinate system, and placed in a single R-tree, and so forth. Examples of operations on RI-trees that will apply on all substructures (e.g. sequence intervals), called *SUB_X*, are represented below:

***ifOverlap*** function: *SUB-X * SUB_X* ->{0, 1}, returns *true* if the two interval substructures overlap.

***Next*** function: *SUB_X* -> *SUB_X* is applicable on data types for which there is a strict ordering on the domain; it returns the sub-structure encountered next in the ordering input substructure. The semantics of "next" depends upon the data types (sequence, anatomical/geographical region, *etc.*).

***Intersect*** function: *SUB_X * SUB_X* -> *SUB_X*, returns the intersection of two *SUB-X*. This operation is valid for convex data types such as sequences and rectangles.

**S1.2 BioNets Ontology**

Any OWL ontology contains a set of OWL classes, their properties and instances. Our OWL schema contains entities (OWL classes) that could be represented as Primary or Graph nodes (Fig. 1C- table Objects and 1D, upper left) in our graph database schema (biological objects and processes such as proteins, genes, and interactions). In addition, it contains a sophisticated hierarchy of attributes (OWL “utility” objects) (Fig 1C table Attribute Value and 1D upper right) that is modified version of attribute hierarchy of the previous version of PathSys graph database [30]. Every data object in our database is associated with elements and relationships of our OWL schema (Fig 1D). Every element in our ontology (Fig 1D) is indexed to preserve the mappings between an external OWL or OBO structured ontologies, in a way that these mappings can be searched themselves, and can be used to associate data objects with the ontologies (or between ontologies). Some of the common operations (e.g. finding k-neighborhood, get ancestors/descendants, find shortest path, etc.) on ontologies implemented in our system are listed below:

*CI : C -> I^+^*, returns the set of all instances of a concept.

*CRI : C x R -> I^+^* returns the set of all instance of a concept by relation R.

*CmRI : C x R^+^ -> I^+^* returns the set of all instances of a concept *c* belonging to *C,* restricted to a set of relation types.

*mCmRI : C^+^ * R^+^ -> I^+^* returns the all the instances reachable via any concepts from a set using only edges from *R^+^*.

*SubTree(X, R1)*: Returns the subtree under X restricted to edge relation R1.

*SubTree(X, R1) - SubTree(Y, R1)*: If Y is a descendent of X, then this operation returns the sub-tree under X minus the sub-tree under Y restricted to relation RI.

BioNets ontology (Fig. S2) consists of the general-purpose Basic Ontology that was manually developed and that maps the classes from different domains; for example, protein, gene, pathway, interaction, disease, cell, tissue, drug, chromosome, COG functional group, gene set (e.g., operon, regulon). Currently, Basic Ontology is manually mapped onto 25 OBO ontologies, including Sequence Ontology, GeneOntology, Human Disease, CheBI, BRENDA Tissues. These 25 ontologies were selected as the ontologies that are curated and regularly updated. Thanks to the efforts of OBO consortium ([www.bioontology.org](http://www.bioontology.org/)) that provides the mapping among more than 200 ontologies, we were able to automatically integrate in the BioNets ontology 98 ontologies total – as new databases will be integrated in our system more ontologies will be added, if needed. The basic.owl file with Basic Ontology and mappings from it to other ontologies can be downloaded at <http://flu.sdsc.edu/bionetsonto.jsp>.


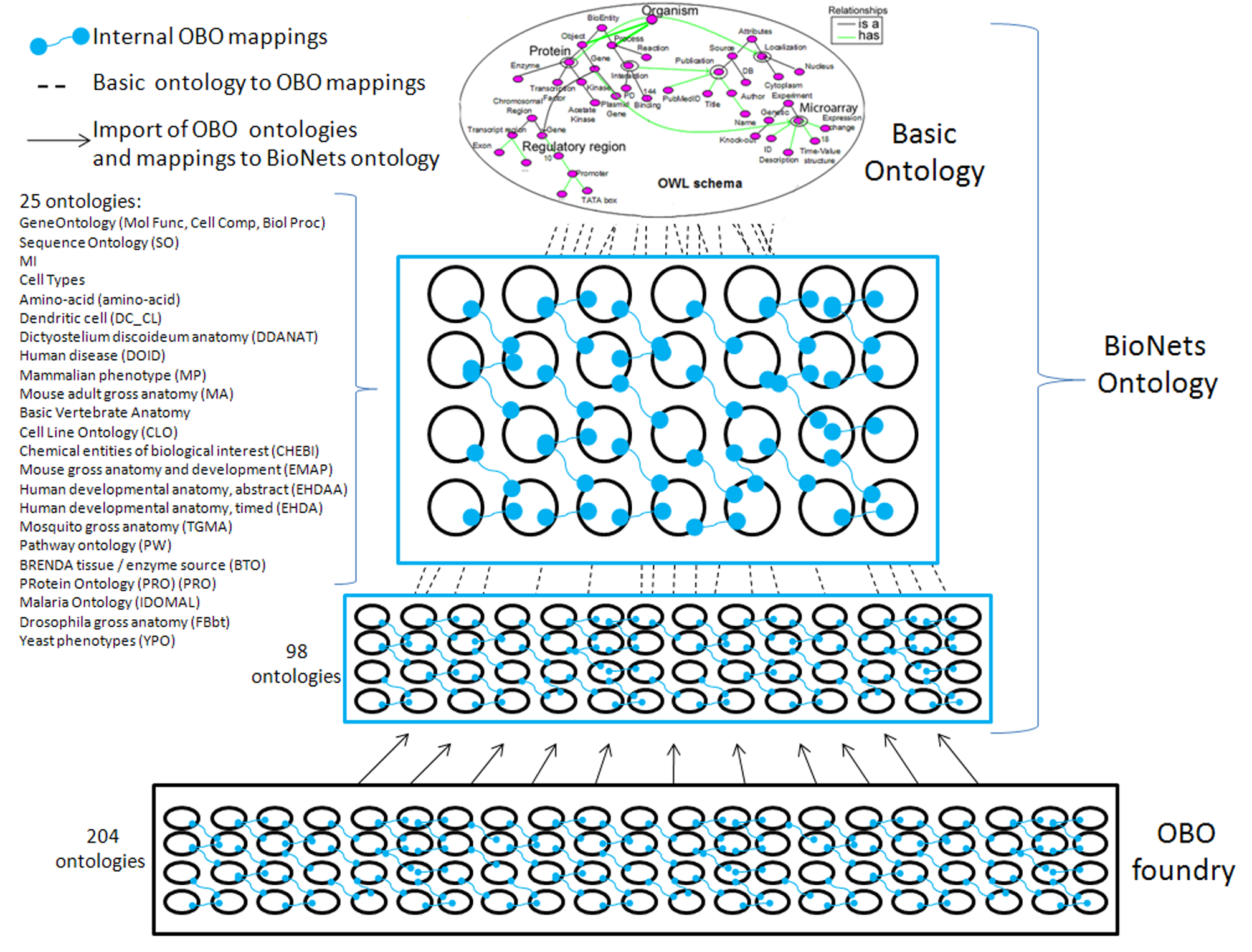


**S2. Schema of integration of OBO ontologies into BioNets ontology.**

We have two kinds of mappings (in the basic.owl):

First, for adding new properties to an existing class (e.g. transcription factor) in an external ontology (e.g. GO Molecular Function) we: 1) create mapping class (e.g. Mapping_GO_0003700) under MappingSupperClass 2) add sameAs property from class Mapping_GO_0003700 to transcription factor using resource url. Thus we can add arbitrary number of properties to classes not modifying external ontology, which is critical for updates.

Second, simply add sameAs property between class in basic and class in external ontology. One can find these sameAs properties under classes: protein, experiment, organ, etc. (in Protégé choose the class and click “Switch to Triples” button).

**S1.3 BioNetQL**

We are developing, *BioNetQL*, query language for heterogeneous data that are connected through interaction networks, ontology graphs and phylogenetic trees and has the flavour of SQL and SPARQL ([www.w3.org/TR/rdf-sparql-query/](http://www.w3.org/TR/rdf-sparql-query/)). SPARQL is a query language of RDF [41] databases that has a graph-structured instance store and graph-structured schema/ontology. It also has the notion of a class hierarchy and a subproperty hierarchy that takes it closer to ontology. Over this data model SPARQL primarily provides an edge pattern language that can return edge sets from RDF instances graphs. SPARQL has an edge model of a graph database, and does not permit path expressions or any other operations that extracts a subgraph whose size is unknown at query time. For such kinds of queries we will use relational querying paradigm.

Our implementation of *BioNetQL* can be queried over mixed (relational and graph structures) data constructs: OWL-like multi-graphs, paths, and trees, sets and bags of nodes, edges and their attributes, but additionally allows the returned values to be bags of paths, trees and graphs. While a complete description of the language and the query evaluation process is beyond the scope of this paper, we present a brief Query Language Syntax description and several features of the language through several examples from our compendium of user operations (see Supplementary File 1 for full list of user’s queries compendium) within and across our four main data types that will drive the development of our Host-Pathogen data management system.

The ability to have functionality for secondary structure querying is crucial for pathogenesis studies. Moreover, it is important to perform both sequence and structure analysis for virulent sequences, described above, because despite the high sequence similarity slight deviations in 3D structure of a mutant virus may cause significant resistance to drugs. Studies of the ill-famous 1918 flu virus [42] have determined the three dimensional surface structures of several proteins of this virus and have been able to delineate structural parameters to be important for lethality. For example, Influenza neuraminidase (N1-N9) is a tetramer of identical subunits of 60 KDa. The subunit fold is the prototypic β-propeller, six four-stranded anti-parallel β-sheets arranged as if on the blades of the propeller. Superposition of the structures of N1, N4 and N8 group-1 neuraminidases reveals that their active sites are virtually identical. However, there are substantial conformational differences between group-1 and group-2 neuraminidases 8,9,17 centered on the ‘150-loop’ (residues 147–152) and the ‘150-cavity’ adjacent to the active site.

**S1.4 Application**

It should be noted that the provided examples of queries are internal BioNetQL queries of the system (Fig. 4) generated in response to the queries constructed using BiologicalNetworks search tools (Fig. 6):

1. Keyword and multi-word search (Fig. 6A)
2. Specialized search (Fig. 6B). The purpose of the specialized is search is to pinpoint mostly used curated data sets (for example GEO Microarrays, Curated Pathways, etc.) for user to quickly and easily find and search these datasets.
3. ‘Comprehensive search by attributes’ (Fig. 6C) (it is located in the upper right corner of the program and depicted by a binocular). The search by attributes allows you to search database objects using many types of data as search conditions. These include, for example, node type, effect (positive, negative, unknown), mechanism (transcription, phosphorylation), tissue type, description/user-defined attributes text, and so forth.

Build Pathway Wizard (Fig. 6D). BuildPathwayWizard (BPW) assists you in finding regulatory paths and functional links, between selected objects, searches for common targets or regulators for the group of molecules, finds connection to Curated Pathways (e.g. KEGG). BPW can find functional links between proteins in the lists imported from other programs (e.g. gene expression clusters).

Query 1:

***Neuraminidase*|*Hemagglutinin*; (localization=inside {nucleus | cytoplasm}):interaction**

Query 2:

We queried our ‘Time/Space/Value dependencies’ type of data (see Fig. 2) containing all microarray experiments from NCBI GEO (as of 10/2009): “select all significant genes in *Mouse* and *Influenza* **microarray_experiments**.”

**Mouse&Influenza;(**

**(expressionChange={overexpressed | underexpressed}):genes**

**):microarray_experiment**


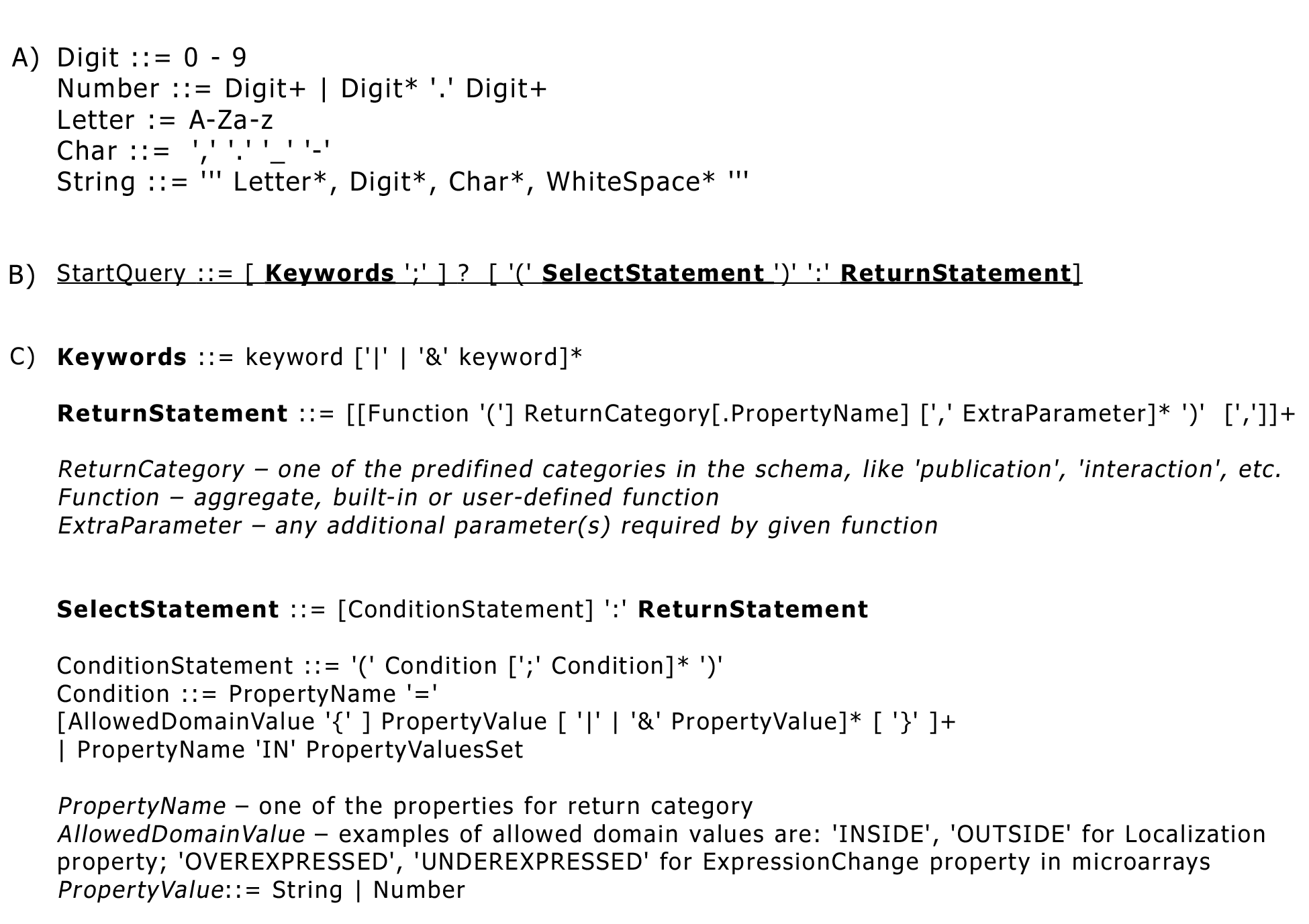
This query returns the list of the experiments (MetaObjects) containing keywords (Mouse and Influenza) in meta-data; every MetaObject contains genes significantly perturbed in that particular experiment. Since different experiments have different number of time points and conditions, data from every experiment were normalized, so that every expression vector is subtracted from the mean and divided by the standard deviation of the experimental expression values. the Pearson correlation calculation is FDR-corrected so that the p-Values calculated for PCC take the length of the expression vectors into account. We treat a gene as up-regulated if its time-averaged expression value is more than twice the average expression value for all genes by all time points in the experiment, i*.e.* :

**S3.** **BioNet query language syntax schema.** **A)** Description of possible literals. **B)** General query string consists of KEYWORDS (0 or more), SELECT and RETURN statements. **C)** ReturnStatement consists of ReturnCategory (e.g. gene, protein, pathway, publication, *etc.*), Function (e.g. get_shortestpath, calculate_correlation, *etc.*) and ExtraParameter. ConditionStatement of the SelectStatment – is a list of Conditions (0 or more), where Condition consists of PropertyName and PropertyValue: for example: TaxID>200 ExpressionChange is OVEREXPRESSED.

*Avg_t (Gene Expression) / Avg_gene_t (Gene Expression) >2*, and as down-regulated if <2

where *Avg_t (Gene Expression) = ∑(GEt)/Nt, Avg_gene_t(Gene Expression) = ∑(∑(GEt)/(Nt))/Ng*

*GEt*- expression value of a gene at time point t

*Nt* – number of time points (in the case of a time series) or number of experiments

*Ng* – number of genes in the microarray experiment

Query 3:

**(expressionChange=underexpressed; experimentCondition=infection):protein**

Query 4:

**:getCoexpressedPairs((expressionChange={overexpressed | underexpressed}; organism=Influenza):gene.geneID)**

Query 5:

**((interactors in {(type='transcription factor'):gene.geneID,**

**(featureType='binding site'):gene.geneID}):interactions AS inters,**

**:findOrthologs('human',‘mouse’,'rat', {inters.interactors }, e-100) ):interaction**

Query 6:

Next we searched for specific known and predicted binding sites: **Which genes are up-regulated by either *Neuraminidase* or *Hemagglutinin* and contain the same binding site with the sequence GGGAAAAA in any regulatory region, in which types of human cells and in response to which signals?**

**signal;(type='human cell';**

**((expressionChange=overexpressed; featureType='binding site';**

**geneID in (:like(NuclSequence, 'GGGAAAAA';**

**:interactsWithSome('*Neuraminidase','Hemagglutinin'*)));**

**):gene.geneID)**

**):metanode**

**Query 7**

Next, using our ‘Comprehensive search by attributes’ tool (Fig 4C) we searched for all human genes/proteins containing in their **attributes** such key**words** as: “influenza, flu, virus, viral, pathogen, etc.” and human genes/proteins mentioned in **publications** containing words like: “influenza, flu, virus, viral, pathogen, etc.”

Second, using Build Pathway Wizard we searched for publications containing genes/proteins from our list of discovered candidates and their interactions/relations: “Find all interactions between Neuraminidase and Hemagglutinin and **publications** in which these **interactions** were published”.

**(interactors =Neuraminidase & Hemaglutinin;interaction.SourceID=publication.ID) :interaction, publication**

This query performs join on two categories – 'publication' and 'interaction' - and returns publication records containing both *Neuraminidase* and *Hemagglutinin* as interactors. Among found genes there are many genes known to be related to anti-viral response mechanism: CREB, HNF1, FOXP3, Pax, Gata factors Stat, Sfrs1.

**References**

[1] Bader G, Betel D, Hogue C: **BIND-The Biomolecular Interaction Network Database.** *Nucleic Acid Res.* 2001, **29**: 242-245.

[2] Lee T, *et al*: **Transcriptional regulatory networks in Saccharomyces cerevisiae.** *Science* 2002, **298**: 799–804.

[3] Uetz P, *et al*: **A comprehensive analysis of protein- protein interactions in Saccharomyces cerevisiae.** *Nature* 2000, **403**:623–627.

[4] Yuryev A: [**In silico pathway analysis: the final frontier towards completely rational drug design**](http://www.informapharmascience.com/doi/abs/10.1517/17460441.3.8.867). *Expert Opinion on Drug Discovery* 2008, V.3(8):867-876

[5] Salah AM: [**Hint to Biomarkers of Acute Aortic Dissection by Pathway Analysis**](http://circres.ahajournals.org/cgi/reprint/103/5/e35)**.** *BCVS Conference 2008*.

[6] Kuznetsov V, Thomas S, Bonchev D: [**Data-driven Networking Reveals 5-Genes Signature for Early Detection of Lung Cancer**](http://doi.ieeecomputersociety.org/10.1109/BMEI.2008.258)**.** bmei,pp. 2008, 413-417.

[7] Hettne K, Cases M, Boyer S and Mestres J: [**Connecting Small Molecules to Nuclear Receptor Pathways**](http://www.ncbi.nlm.nih.gov/sites/entrez?Db=pubmed&Cmd=TermToSearch=17897040)**.** *Curr Top Med Chem.* 2007, 7(15):1530-6

[8] Yuryev A: **In silico pathway analysis: the final frontier towards completely rational drug design**. *Expert Opin on Drug Discov*. 2008, 3(8):867-876

[9] Sivachenko A, Kalinin A, Yuryev A: [**Pathway analysis for design of promiscuous drugs and selective drug mixtures.**](http://www.ingentaconnect.com/content/ben/cddt/2006/00000003/00000004/art00003) *Curr Drug Discov Technol*. 2006, Dec;3(4):269-77.

[10] Sivachenko A, and Yuryev A: [**Pathway analysis software as a tool for drug target selection, prioritization and validation of drug mechanism**](http://www.expertopin.com/doi/abs/10.1517/14728222.11.3.411)**.** *Expert Opinion on Therapeutic Targets*, v.11(3), pp.411-421

[11] Frykns M, Rickardson L, Wickstrm M, Dhar S, Lvborg H, Gullbo J, Nygren P, Gustafsson MG, Isaksson A, and Larsson R: [**Phenotype-Based Screening of Mechanistically Annotated Compounds in Combination with Gene Expression and Pathway Analysis Identifies Candidate Drug Targets in a Human Squamous Carcinoma Cell Model**](http://jbx.sagepub.com/cgi/content/abstract/11/5/457)**.** *J Biomol Screen.*, 11: 457 - 468,

[12] Chanan-Khan AA, Padmanabhan S, Stein L, Panzarella J, Miller KC, and Hawthorne L: [**Validating Molecular Targets of Thalidomide in CLL: Net Effect of Increased Apoptosis through the Intrinsic Pathway and down Regulation of NF-kB Signaling- Validation Using Gene Expression Profile from the Phase I/II Clinical Trial of Thalidomide and Fludarabine.**](http://meeting.bloodjournal.org/cgi/content/abstract/106/11/5043?maxtoshow=&HITS=&hits=&RESULTFORMAT=1&andorexacttitle=and&fulltext="pathway+assist"&andorexactfulltext=and&searchid=1135271543558_15421&FIRSTINDEX=0&sortspec=relevance&resourcetype=1) *Blood*, ASH Annual Meeting Abstracts 2005, 106: 5043.

[13] Good BM, Wilkinson MD: **The Life Sciences Semantic Web is full of creeps!** *Brief Bioinform* 2006, **7**(3): 275-286
